# The motivations and methodology for high-throughput PET imaging of small animals in cancer research

**DOI:** 10.1007/s00259-012-2177-x

**Published:** 2012-07-13

**Authors:** Nicolas Aide, Eric P. Visser, Stéphanie Lheureux, Natacha Heutte, Istvan Szanda, Rodney J. Hicks

**Affiliations:** 1 Nuclear Medicine Department, François Baclesse Cancer Centre, Avenue Général Harris, 14076 Caen Cedex, France; 2 BioTICLA team, EA 4656, IFR 146, Caen University, Caen, France; 3 Nuclear Medicine Department, Radboud University Nijmegen Medical Center, Nijmegen, The Netherlands; 4 Clinical Research Unit, François Baclesse Cancer Centre, Caen, France; 5 Division of Imaging Sciences and Biomedical Engineering, King’s College London, London, UK; 6 Centre for Molecular Imaging, Peter MacCallum Cancer Centre, East Melbourne, Australia

**Keywords:** High throughput, Sample size, Clinical PET/CT, Small-animal PET, Planar positron imaging systems, NEMA NU 4, Animal models

## Abstract

Over the last decade, small-animal PET imaging has become a vital platform technology in cancer research. With the development of molecularly targeted therapies and drug combinations requiring evaluation of different schedules, the number of animals to be imaged within a PET experiment has increased. This paper describes experimental design requirements to reach statistical significance, based on the expected change in tracer uptake in treated animals as compared to the control group, the number of groups that will be imaged, and the expected intra-animal variability for a given tracer. We also review how high-throughput studies can be performed in dedicated small-animal PET, high-resolution clinical PET systems and planar positron imaging systems by imaging more than one animal simultaneously. Customized beds designed to image more than one animal in large-bore small-animal PET scanners are described. Physics issues related to the presence of several rodents within the field of view (i.e. deterioration of spatial resolution and sensitivity as the radial and the axial offsets increase, respectively, as well as a larger effect of attenuation and the number of scatter events), which can be assessed by using the NEMA NU 4 image quality phantom, are detailed.

## Why we will need to image many animals within a single PET experiment

The number of animals that require to be imaged within a single small-animal PET (SA-PET) experiment is driven by the number of groups planned for the experiment, including controls and treatment arms. As opposed to initial SA-PET studies where the effect of conventional chemotherapy on tracer uptake in tumours was tested at the maximum tolerated dose against a control group, the recent development of molecularly targeted therapies can potentially lead to a dramatic increase in the number of groups to be imaged. This is driven by the concept of the optimal biological dose (OBD) that, for such targeted therapies, may be well below and more relevant than the maximum tolerated dose. OBD, defined as the dose that achieves a target plasma concentration or reliably inhibits a drug target, might be established based on pharmacokinetic endpoints and ideally on pharmacodynamic assays by demonstrating directly the biological effect on the target and its downstream molecules in normal or tumour tissues [1–3]. In case of the pharmacokinetic endpoint, it has to be shown that the target concentration chosen can inhibit the drug target in the tumour. This requires accounting for plasma protein binding, which determines the amount of free drug available to interact with the target, as well as interindividual variations in drug absorption and metabolism. When target modulation is chosen as the endpoint, the drug targets as well as the required magnitude of inhibition have to be known. An OBD should inhibit the identified target in tumours, but most importantly there should be evidence that modulating the target in tumours consistently leads to growth inhibition. In some cases this strategy may fail, as shown by Fuereder et al. [4] who demonstrated that the in vivo sensitivity of gastric cancer xenografts to BEZ235, a dual phosphoinositidine 3-kinase (PI3K)/mTOR inhibitor, does not correlate with in vitro antiproliferative activity or in vivo PI3K/mTOR target inhibition by BEZ235. In contrast, ^18^F-FLT uptake was significantly decreased, as compared to control, in a cell line sensitive to BEZ235, suggesting that ^18^F-FLT PET could be used as a surrogate marker in clinical trials evaluating dual PI3K/mTOR inhibitors.

In the context of OBD definition, SA-PET imaging is able to screen a large library of chemicals for their ability to modulate the identified target without the requirement for tissue sampling and to assess delivery and dose issues in preclinical studies, thus helping in planning the development of later phases [5]. Cejka et al. [6] have shown that FDG uptake can be used as a surrogate marker for defining the OBD of a molecularly targeted therapy. In their study, Cejka et al. [6] showed that everolimus blood levels increase in a dose-dependant manner but antitumour activity, as assessed by ^18^F-FDG uptake, reaches a plateau at a defined dose level. In the context of cancer research and preclinical drug development, the number of animals to be imaged to define such a dose–response curve is potentially high. The challenge is increased when designing optimal combinations of targeted therapy and standard chemotherapy, and the most effective scheduling of drug administrations, for example whether targeted therapy should be used concurrently, before or after the standard chemotherapy. An improvement in throughput in SA-PET imaging would allow the evaluation within a single PET experiment of all the criteria mentioned above and would affect the cost, logistics and speed of preclinical studies.

The number of animals to be scanned to reach statistically significant differences can be computed. For that purpose, one needs to choose an appropriate test and to know the expected decrease in tracer uptake in the treated animals as compared to the control animals, the number of groups that will be imaged, and the expected intra-animal variability for a given tracer. This work has already been done by Eckelman et al. [7, 8]. However, these authors based their calculations on the imaging of only two groups (i.e. control group and treated animals). Table 1 summarizes the required number of animals in each group, for up to five groups. The coefficient of variation (CV) for tracer uptake was set at 15 % based on the results of studies by researchers at Stanford University who imaged tumour-bearing mice twice on the same day after injection and reinjection of ^18^F-FDG [9], ^18^F-FLT [10] or ^18^F-labelled RGD [11] and determined the CV for standardized uptake value and/or percent injected dose per gram (%ID/g) as the ratio between standard deviation of the two measurements and their mean. The mean CVs were 15.4 %, 14 % and 10.5 % for ^18^F-FDG [9], ^18^F-FLT [10] and ^18^F-labelled RGD, respectively [11]. These authors showed that the observed CVs were likely to have been due to the sizes of the volumes of interest (VOI). Indeed, when redrawing VOIs 2 months apart, it was shown that CVs for %ID_mean_/g between the original and redrawn VOIs were 6.5 ± 4.7 % and 6.6 ± 3.9 % for the ^18^F-FLT study and the ^18^F-labelled RGD study, respectively. In a second analysis in which VOIs of the same size were used to assess VOI placement, the CVs were much lower (2.3 ± 1.5 % and 2.1 ± 1.5 % for the ^18^F-FLT study and the ^18^F-labelled RGD study, respectively). These results suggest that the variability in the %ID_mean_/g was largely a result of the VOI size rather than VOI placement. Other technical factors including the efficiency of tail vein injection, volume of injected tracer and injected activity had little or no influence on the observed CVs. For ^18^F-labelled RGD, animal handling had no influence on the CVs, while it was shown to have great importance in the ^18^F-FDG and ^18^F-FLT studies, with the addition of the thymidine kinase blood activity for ^18^F-FLT. The issue of animal handling when imaging multiple mice is further discussed in section entitled “Issues related to animal handling”. A hypotheses tested included decreases in tracer uptake ranging from 25 % to 75 %, as molecularly targeted therapies can lead to variable tumour responses [4, 12] and also the observed response for a given treatment may be different for tracers associated with different biological pathways such as ^18^F-FDG and ^18^F-FLT [13]. As shown in Table 1, as many as 45 mice would be needed for an experiment in which five groups of mice are to be imaged and a 30 % decrease in tracer uptake is expected.

**Table 1:** Number of animals per group required for imaging to reach statistical significance in relation to the number of groups and the percentage decrease in tumour uptake. Data were computed for a two-tailed unpaired Student’s *t*-test (α risk 5 %, 1 − β power 80 %), assuming equality of variance and normal distributions, with Bonferroni correction for multiple comparisons

Change in %ID/g (%)	No. of groups							
	2		3		4		5	
	No. of mice per group	No. of possible comparisons between groups^a^	No. of mice per group	No. of possible comparisons between groups^a^	No. of mice per group	No. of possible comparisons between groups^a^	No. of mice per group	No. of possible comparisons between groups^a^
25	7	1	9	3	11	6	12	10
30	6	1	7	3	8	6	9	10
50	3	1	4	3	5	6	5	10
75	3	1	3	3	4	6	4	10

^a^Between control and treatment groups and between treatment groups when different doses or schedules are evaluated.

## Challenges in achieving high-throughput PET studies

### Physics issues

An obvious means of increasing the throughput of SA-PET studies is to image several animals simultaneously. This can be achieved with high-resolution clinical PET, large-bore dedicated SA-PET systems or large field-of-view (FOV) planar positron imaging systems. However, this potentially leads to issues with the physics of imaging. Indeed, scanning multiple animals simultaneously would be expected to reduce image quality for the following reasons. First, spatial resolution and sensitivity deteriorate as the radial and the axial offsets increase, respectively. Second, the presence of more than one source of radioactivity increases Poisson noise, the effect of attenuation and the number of scatter events. Although attenuation and scatter corrections are available, a large attenuating mass within the FOV will lower the total number of detected true coincidences per mouse and it is unsure whether scatter correction algorithms perform well in the case of multiple and noncentred sources.

**Fig. 1:**
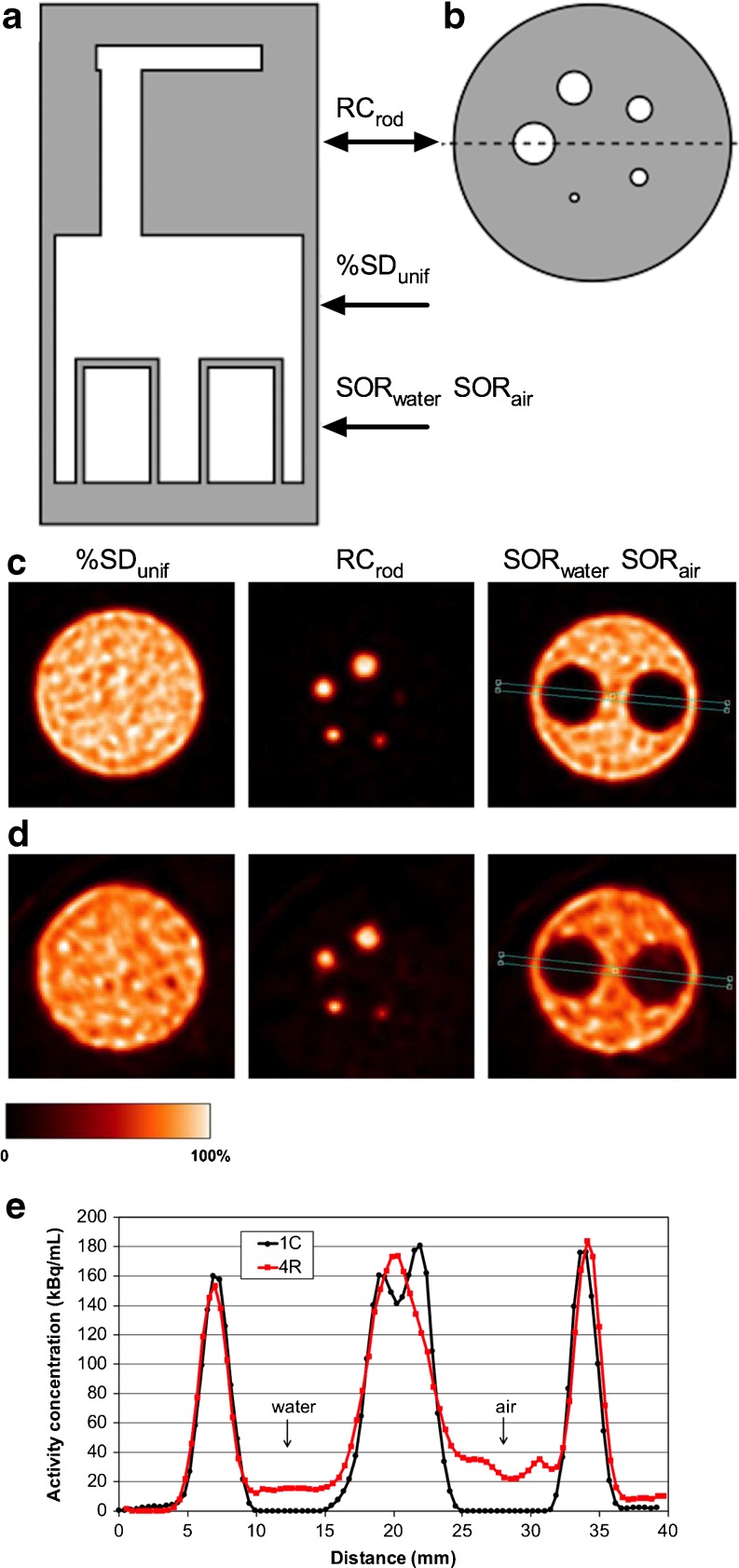
Diagram of the NEMA NU 4 image-quality (NEMA NU 4) phantom (**a**, **b**) and illustration of the impact of radial displacement on image quality parameters. Cross-sectional diagram of NU 4-IQ phantom. *Grey* represents solid polymethylmethacrylate, and *white* represents hollow, fillable compartments (rods ranging in diameter from 1 to 5 mm, a uniform region that is filled with an ^18^F solution, and water- or air-filled cylinders). Views are coronal (**a**) and transverse through the rods (**b**). **c**, **d** Transaxial images of three regions of interest (%STD_unif_ percent standard deviation in the uniform region; *RC*_*rod*_ recovery coefficients, ; *SOR*_*water*_ and *SOR*_*air*_ spillover ratios in water and air) in the NU 4-IQ phantom imaged alone at the centre of the FOV (1C position, **c**) or together with three additional scatter/attenuation sources with a 20.3 mm radial displacement (4R position, **d**). Data were acquired on an Inveon SA-PET scanner and reconstructed using MAP. Larger values for SD_unif_, SOR_water_ and SOR_air_ and smaller RC values for the 1mm rod can be visually noted. **e** Cross-sectional profiles through the nonradioactive compartments demonstrate the difference in SOR_water_ and SOR_air_ imaging the phantom in either the 1C or 4R position. The images correspond to the central transaxial planes through the regions. **c**, **d** and **e** have been adapted and reprinted with permission (Siepel et al. [33])

When evaluating a configuration where multiple animals are to be imaged within the FOV of a PET scanner, the physics issues mentioned above could be addressed with the NEMA NU 4 image-quality phantom (Fig. 1). Briefly, this phantom consists of the following three regions: a main fillable uniform region chamber, a lid that attaches to the main fillable region containing two smaller cold region chambers one filled with nonradioactive water and the other with air, and a solid acrylic glass region with five fillable rods drilled through with diameters of 1, 2, 3, 4 and 5 mm. Following a 20-min emission scan using ^18^F, image quality is assessed as follows: (a) image noise is expressed as the percentage standard deviation in a central, cylindrical VOI drawn over the uniform region of the phantom, (b) recovery coefficients (RCs) for the filled rods are expressed as the ratio between the maximum activity concentration measured in the rods divided by the mean activity concentration in the uniform region of the phantom, and (c) the spillover ratios are expressed as the ratios of the mean activity in the water- and air-filled compartments divided by the mean activity concentration in the uniform region of the phantom.

Examples of the imaging of multiple mice with different systems are discussed below. Apart from the report of Aide et al. [14] of dynamic studies with ^68^Ga-EDTA in a group of three mice imaged simultaneously, there are no published data on non ^18^F positron emitters using multiple animals in the FOV. It can be expected that for these positron emitters, stronger photon attenuation (animals radially displaced next to each other) and loss of sensitivity (animals placed behind each other, displaced axially) occurs in the same way as for ^18^F. Specific problems that have to be dealt with using several of these positron emitters are (1) the longer positron range than with ^18^F leading to a deterioration of spatial resolution, and (2) additional single photons (especially when present in the 511-keV energy window, such as with ^124^I) that give rise to additional, not properly localized events resulting in an additional, more or less uniform background concentration that can affect both image quality and quantitative accuracy [15, 16]. Accordingly, Disselhorst et al. [15] imaged the NEMA NU 4-IQ phantom with ^18^F, ^68^Ga, ^124^I and ^89^Zr, and found that radionuclides with large positron ranges (^68^Ga, ^124^I) had smaller RCs (for the same rod diameter) than those with short ranges (^18^F, ^89^Zr). However, single photons only slightly affected %STD_unif_ and SOR_air_ (that are mainly affected by scattered and single photons), which were roughly the same for all four radionuclides. SOR_water_, on the contrary, was larger for ^68^Ga and ^124^I, but as explained Disselhorst et al. [15], this could be attributed to their longer positron range that leads to positrons being emitted in the body part of the phantom but annihilated in the cold water compartment.

As the activity in the FOV increases (as in imaging multiple mice simultaneously), increasing numbers of random coincidences will occur. A way of improving image quality with nonstandard positron emitters is to optimize acquisition settings [17] and to use advanced reconstruction algorithms with positron range modelling [18, 19].

### Clinical PET/CT systems equipped with new reconstruction algorithms

Tatsumi et al. [20] investigated the feasibility of imaging rodents with a clinical PET scanner (a General Electric Discovery LS tomograph with a 5 mm spatial resolution) using ex vivo counting as the reference standard. They showed that imaging tumours was feasible in rats and rabbits, but image quality in mice was lower because of their smaller size. These findings were later confirmed by Seemann et al. [21] who compared quantitative data from tumour-bearing mice imaged on a Siemens Biograph PET/CT scanner and on a SA-PET scanner (Mosaic, Philips Medical Systems). They found that tumours imaged by SA-PET had a 1.89 higher mean tumour/background ratio than those imaged by PET/CT because of significant partial volume effects related to the inferior spatial resolution of the clinical PET/CT scanner.

**Fig. 2:**
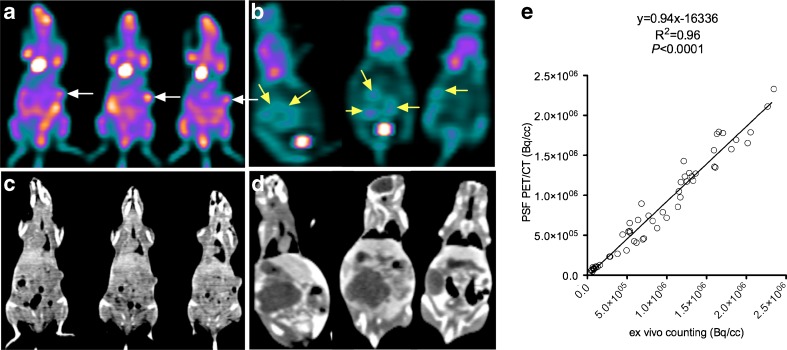
High-resolution clinical PET/CT images of tumour-bearing mice imaged in a group of three. **a**, **b** Representative PET coronal slices in mice bearing subcutaneous tumours (**a**) or ovarian tumours (**b**) displaying heterogeneous ^18^F-FDG uptake are shown. **c**, **d** Corresponding CT coronal slices. **e** Linear regression between PET quantitative data (pooled data from Aide et al. [14, 23], including 33 organs and 22 tumours) and ex vivo counting shows an excellent correlation with a slope almost equal to unity. The PET data were reconstructed with an iterative algorithm that models the PSF of the PET system

The impact of the use of scanner characteristics in the process of iterative image reconstruction in order to image small animals on a clinical PET scanner was first described by Brix et al. [22]. These authors showed that the spatial resolution at the centre of the FOV could be improved from 6.5 mm with standard ordered subsets expectation maximization to 3.9 mm with an iterative reconstruction method incorporating the spatially variant point spread function (PSF) of the scanner. In two more recent studies [14, 23] a commercially-available PET/CT device equipped with PSF reconstruction was used to image several mice simultaneously. The spatial resolution of this system measured according to NEMA NU-2001 standards is 2.09 mm at the centre of the FOV [14]. In these studies in which mice were imaged in groups of three or four after injection of ^18^F-FDG, ^18^F-FLT or ^68^Ga-EDTA, it was shown that PSF PET/CT can provide good quality images and accurate quantification of the radioactivity within the imaged lesions of mice bearing tumours and has the capacity to simultaneously image multiple mice in static or dynamic mode. Pooled data from these two studies are shown in Fig. 2.

 PSF PET imaging of mice can be further improved by “super-resolution”, a technique using stepped images that are postprocessed with iterative deconvolution, as shown by DiFilippo et al. [24]. These authors used an apparatus equipped with three motorized stages and an animal bed on a Siemens mCT system, whose physical performances have been described in details elsewhere [25]. DiFilippo et al. [24] performed 16-min acquisitions by stepping the NEMA NU 4 phantom or animals at 1-min intervals in the three orthogonal directions. They showed an improvement in organ delineation and RCs for the 2-mm rod of the NEMA NU 4 phantom of 0.21 and 0.39 for PSF images and PSF super-resolution images, respectively. This can be compared to RC values of 0.48, 0.58 and 0.38 for the Siemens Inveon scanner [26], the Bioscan/Mediso Nano PET/CT scanner [27] and the GE FLEX Triumph scanner [28], respectively.

 Revheim et al. [29] imaged ten mice simultaneously, and demonstrated the feasibility of a clinical PET/CT scanner for monitoring the effects of molecularly targeted therapies on gastrointestinal stromal tumour xenografts.

Although clinical PET/CT may be used in centres where a dedicated SA-PET is not available and where experiments require a high throughput, one should note that the main drawback of using a clinical PET/CT device for the imaging of mice is its sensitivity, which is much lower than that of a dedicated SA-PET scanner. Indeed, dedicated SA-PET scanners have much larger aspect ratios (axial length/detector ring diameter), leading to higher sensitivity. Although the aspect ratio of the clinical Siemens PET device used by Aide et al. [14, 23] is 0.26 due to its extended axial FOV of 21.6 cm (clinical PET scanners usually have an aspect ratio in the order of 0.2), it is still much lower than aspect ratio of dedicated SA-PET scanners which have aspect ratios up to 0.8 (Siemens Inveon [26, 30]).

### Large-bore SA-PET systems

SA-PET devices with large axial and transaxial FOVs theoretically allow several animals to be imaged simultaneously. The bed provided by manufacturers is generally suitable for scanning two mice simultaneously, one behind the other (depending on the axial FOV of the SA-PET scanner) or side by side, as previously reported by Paproski et al. [31] who imaged mice bearing tumours with ^18^F-FLT on a Siemens microPET R4 scanner. However, imaging more than two mice simultaneously requires a customized bed to be designed. In this regard, Aide et al. [32] and Siepel et al. [33] simulated experiments with multiple mice by using the NEMA NU 4 image quality phantom at different positions within the FOV and adding up to three “mouse phantoms”, i.e. vials containing the same activity concentration as used in the NEMA NU 4 phantom and mimicking attenuation and scatter events. In both studies, a Siemens Inveon SA-PET scanner was used. This device has axial and transaxial FOVs of 126 and 110 mm, respectively.

Siepel et al. [33] imaged the NEMA NU 4 phantom along with three mouse phantoms with all phantoms displaced radially (20.3 mm displacement) or in a combination of radial displacement (22.6 mm) and axial displacement (22.4 mm). They demonstrated that for scanning four mice, combined axial and radial displacement is preferable to just radial displacement, as the latter leads to higher noise and spillover ratios and lower RCs. Figure 1 shows the extent to which NEMA NEMA NU 4 image quality parameters worsen when imaging the NU 4-IQ phantom together with three additional scatter/attenuation sources with radial displacement. In their study in which phantoms and mice were displaced radially (22 mm) only, Aide et al. [32] also found that radial displacement led to a decrease in RCs and an increase in spillover ratios as compared to a single phantom acquisition. They also imaged mice bearing pertinent-sized tumours (22 tumours, median volume 150 mm^3^) and, using ex vivo counting as the reference standard, obtained accurate quantitative values (*r* ^2^ = 0.91, *P* < 0.0001). They also found no difference in accuracy of quantitative values for external tumours (i.e. those located close to the edge of the FOV, where the loss in spatial resolution would be expected to be more pronounced) and those located closer to the centre of the FOV.

**Fig. 3:**
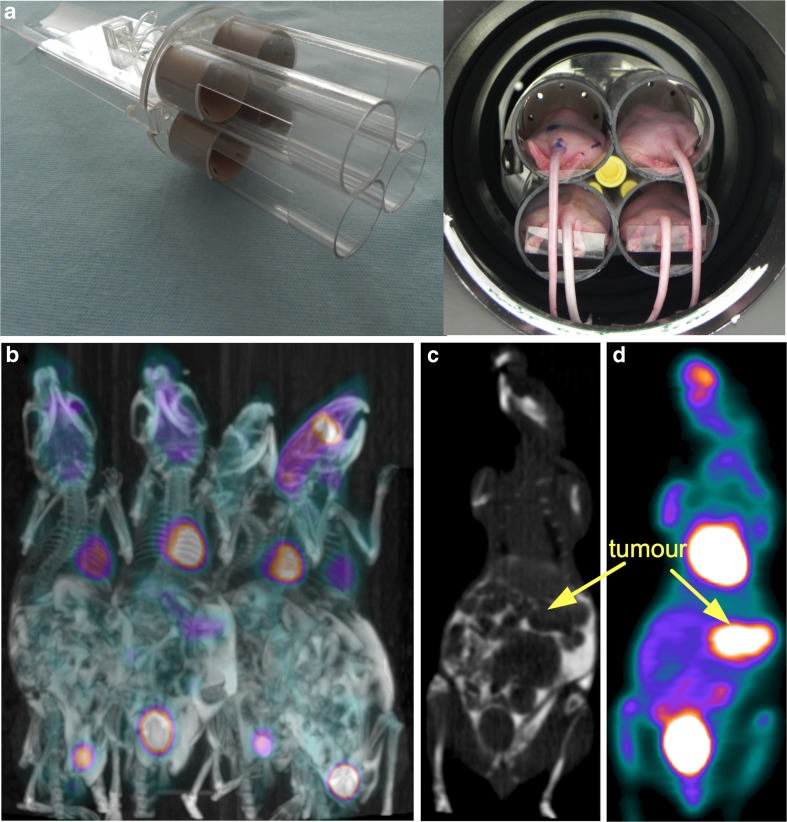
A customized bed designed to image four mice simultaneously with radial displacement. **a** Animals are placed in cylinders (inner diameter 35 mm, leading to a 20-mm radial displacement) that deliver isoflurane for anaesthesia. **b** Fused ^18^F-FDG PET/CT MIP view of four mice bearing abdominal tumours that received an intraperitoneal injection of iodinated contrast medium plus an intravenous injection of Fenestra VC, a long-lasting contrast medium. **c**, **d** Representative coronal slices from contrast-enhanced CT and PET scans at the level of an abdominal tumour (*arrows*)

**Fig. 4:**
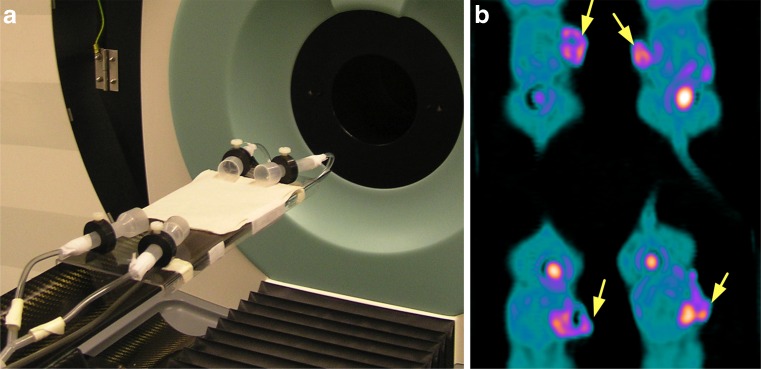
A customized bed designed to image four mice simultaneously with a combination of radial and axial displacement. **a** Animals are placed on a customized bed that delivers isoflurane for anaesthesia and is attached to the manufacturer’s bed. Mice are imaged with an axial displacement of 40.1 mm and a radial displacement of 22.5 mm. **b** Coronal ^18^F-FLT PET image of four mice bearing subcutaneous tumours (*arrows*)

Figures 3 and 4 illustrate the use of customized beds in a large-bore SA-PET system (Siemens Inveon) with either radial displacement or a combination of axial and radial displacement. The PET systems illustrated in these figures use either external sources or CT for attenuation correction. CT-based attenuation correction has the advantage of being faster and inducing less noise than external sources in the final reconstructed images [34], and also provides anatomic referencing, as shown in Fig. 3. For subcutaneous xenograft models, it also offers the advantage of providing more accurate tumour volume evaluation than measurement with callipers [35]. However, CT can deliver high radiation doses causing significant DNA damage and therefore potentially confounding experimental outcome. In that setting, Kersemans et al. [36] showed that by reducing x-ray voltage, flux and duration, it is possible to significantly reduce radiation burden to the animal while maintaining image quality.

**Fig. 5:**
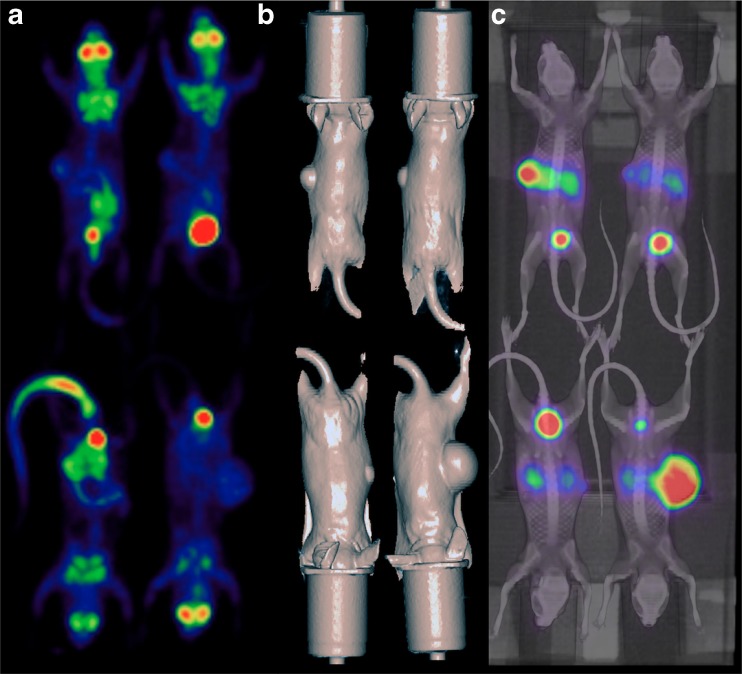
Imaging of multiple mice with a positron emitter other than ^18^F. Four mice with implanted A247 tumours (small axis ranging from 4.6 mm to 12 mm) transfected to overexpress SSTR subclass 2 receptors (cells courtesy of Buck Rogers, Washington University, MI) were imaged 24 h apart on a Mosaic SA-PET system 1.5 h after injection of ^18^F-FDG (mean activity per mouse 8 ± 0.1 MBq) and ^68^Ga-DOTATATE (mean activity per mouse 13.2 ± 0.2 MBq). The animals were scanned with a combination of radial (18 mm) and axial (57 mm) displacement. **a** MIP views for ^18^F-FDG. **b** Given the lack of anatomical landmarks on ^68^Ga-DOTATATE SA-PET images, mice were scanned on a clinical CT scanner and surface images extracted from the CT images. **c** Fused SA-PET/CT MIP ^68^Ga-DOTATATE images (SA-PET images are courtesy of David Binns and Carleen Cullinane, Peter MacCallum Cancer Centre, Melbourne, Australia)

Figure 5 shows the impact of the use of long-range positron emitters on image quality when imaging four mice with a combination of radial and axial displacement in a Phillips Mosaic system [37].

Overall, image quality and quantitative accuracy depend on the size of the scanner's FOV, the size and number of animals imaged and their position within the FOV, and the performance of reconstruction and correction algorithms. Regarding positioning of the animals within the FOV, customized beds should be designed so that regions of interest are imaged as close as possible to the centre of the FOV. Table 2 summarizes results in terms of spatial resolutions and sensitivities at the centre of the FOV with 2- and 3-cm radial offsets for three commercially available large-bore SA-PET devices [26–28, 30, 38]. For instance, when imaging mice with a combination of 3 cm radial and 3 cm axial displacement, one would expect a deterioration of radial full-width at half-maximum (FWHM) due to radial displacement ranging from 24 % to 46 % for filtered back-projection (FBP) and from 11 % to 33 % for advanced reconstruction algorithms. An expected loss of sensitivity due to axial displacement would also be observed, ranging from 48 % to 61 %.

**Table 2:** Impact of radial offset on spatial resolution and of axial offset on sensitivity of commercially available scanners

Scanner	Reference	Reconstruction algorithm	FWHM (mm)^a^									Sensitivity (%)^a^		
			Centre of FOV			2 cm radial offset			3 cm radial offset			Centre of FOV	2 cm axial offset	3 cm axial offset
			Tangential	Radial	Axial	Tangential	Radial	Axial	Tangential	Radial	Axial			
Triumph LabPET-8	[38]	FBP^a^	1.8	1.7	2.4	1.7	2.3	2.5	1.7	2.7	2.7	1.3	0.9	0.5
		MLEM^b^	1.0	0.8	1.6	1.2	1.1	1.5	1.3	1.2	1.5			
Inveon	[26, 30]	FBP^a^	1.5	1.6	2.0	1.5	2.1	2.4	1.6	2.1	2.4	10.1	6.9	5.3
		OSEM/MAP^c^	1.6	1.6	1.7	1.6	1.7	1.6	1.7	1.8	1.6			
NanoPET/CT	[27]	FBP^a^	1.15	1.38	1.47	1.64	2.04	1.6	1.81	2.54	1.89	7.7	4.8	3.1
		3-D OSEM^d^	0.59	0.72	0.60	0.71	0.90	0.66	0.78	1.06	0.65			

*FBP* filtered back-projection, *MLEM* maximum likelihood expectation maximization, *OSEM* ordered subsets expectation maximization, *MAP* maximum a posteriori.

Data for the Triumph LabPET-8 and the Inveon systems were interpolated from Prasad et al. [38] and Visser et al. [30], respectively, and data for the NanoPET/CT system were calculated for the purposes of this paper.

^a^Data obtained as per NEMA protocol.

^b^Ten iterations.

^c^18 iterations/16 subsets, uniformity constraint set to “resolution”, smoothing parameter β = 0.5; all MAP reconstructions were preceded by two 3-D OSEM iterations.

^d^20 iterations/1 subset, no postfiltering.

The results shown in Table 2 are measures of detector performance and are not necessarily indicative of final image quality after postprocessing. They are therefore not meant to be used as a comparison between the three scanners. FWHM are given as determined from FBP as per NEMA NU-A standards. While this algorithm is rarely used in routine practice for rodent imaging, it allows the evaluation of the effect of radial offset on spatial resolution because of hardware issues, i.e. oblique penetration of photons at the edge at the FOV causing parallax error. As shown in Table 2, iterative reconstruction methods provide better FWHM at the centre of the FOV, and also 3-D maximum a posteriori (MAP), which models the PSF of the system [39, 40], is less sensitive to radial offset displacement. However, ideally, the impact of such algorithms in the imaging of multiple mice as well as their optimization should be evaluated with the NEMA NU 4 image quality phantom [41]. For example, increasing the number of iteration will increase the spatial resolution (assessed by RC measurements) but will increase the image noise (as assessed by %STD).

When imaging four mice simultaneously in a combination of radial and axial displacement, whole-body scanning will not always be feasible because of practical issues related to the size of the animals and of anaesthesia devices. Moreover, the useful FOVs of the PET device may be reduced by the CT component. For the Inveon SA-PET/CT scanner, the axial and transaxial FOVs are both reduced to 85.7 mm, as compared to 126 and 110 mm, respectively, for the stand-alone SA-PET scanner. If whole-body scanning is required, for instance for biodistribution studies, the configuration in which all mice are displaced radially would be preferred.

When designing a customized bed, the choice of materials should take into account how difficult they are to manufacture and their attenuating properties, especially if the SA-PET scanner is not equipped with an external transmission source or with a docked microCT scanner for attenuation correction purposes.

### Planar positron imaging and partial ring SA-PET systems

Low-cost planar positron imaging systems have been developed by several groups [42, 43]. These devices have two planar detectors placed facing each other, and are kept stationary during the acquisitions. Because of the limited angle tomography, coronal images obtained with the planar positron imaging systems first developed were similar to projection images created by summing all radioactivities along a depth direction of an object. Therefore, when measuring tumour activity in a subcutaneous lesion, the radioactivity in nontumour tissues that surround the actual tumour has to be subtracted from the gross activity to obtain the net activity in the tumour. A potential disadvantage of these devices is that they do not include attenuation or scatter corrections. More recently, PETbox [44], a planar positron imaging system equipped with an iterative reconstruction method with the incorporation of a system probability matrix has been developed to reconstruct 3-D images from the limited angle projection data.

**Fig. 6:**
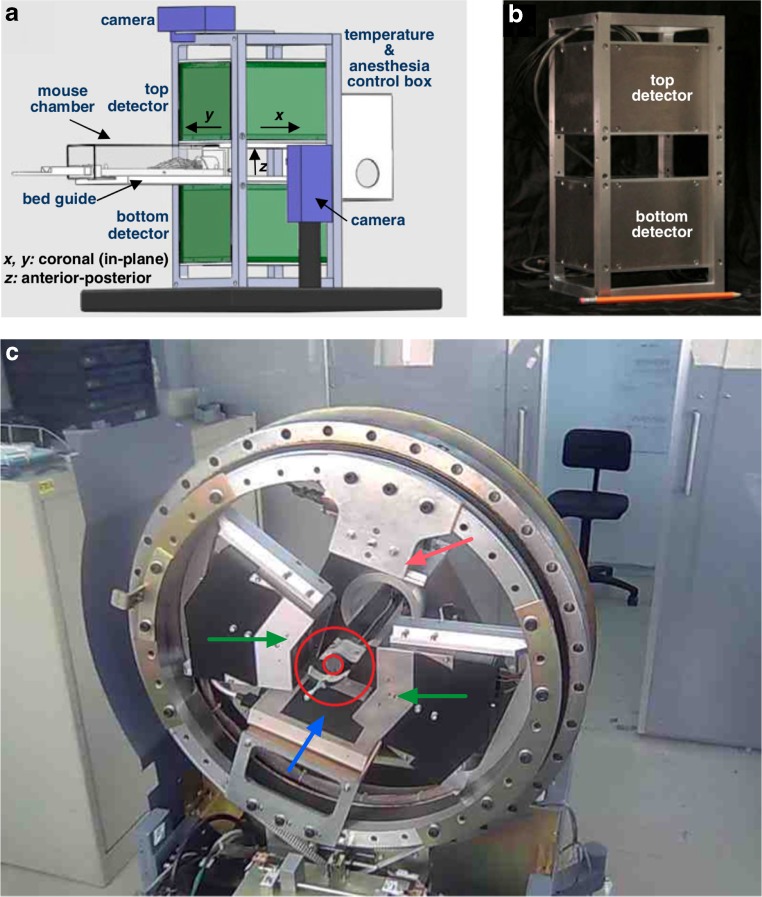
Planar and V-shaped positron imaging systems. **a**, **b** PETbox system [44]: schematic illustration in a bench top configuration (**a**) and photograph of the gantry with the two detector heads assembled (**b**). The two detectors are placed facing each other at a spacing of 5 cm and are kept stationary during the scan, forming a dual-head geometry optimized for imaging mice. **c** VrPET/CT system [45], a multimodality scanner with coplanar geometry. The PET component consists of four detectors arranged in two V-shaped blocks, and the PET and CT components are assembled on a rotating gantry in such a way that there is no axial displacement between the geometric centres of the two modalities. The *small red circle* indicates a NEMA mouse-sized (25 mm diameter) cylinder and the *larger red circle* indicates the transaxial FOV of the scanner (86.6 mm) that is suitable for imaging multiple mice. Also visible are the flat panel x-ray detector (*blue arrow*), the x-ray tube (*red arrow*) and the V-shaped PET detectors (*green arrows*). **a** and **b** reprinted with permission from Zhang et al. [44] and **c** courtesy of Dr. Eduardo Lage, Hospital General Universitario Gregorio Maranon, Madrid, Spain

 Efforts to improve system sensitivity and to aid animal handling (with regard to anaesthesia, temperature control and reproducible optimal positioning of the mice in the FOV) are being made and could be considered as alternative mechanisms to enhance throughput. Moreover, some systems have a sensitive area that is large enough to be suitable for the imaging of two mice simultaneously. Also available is a VrPET/CT scanner [45], a multimodality scanner with coplanar geometry and a 86.6-mm transaxial FOV that would also be suitable for imaging two mice simultaneously. The PET component of this system consists of four detectors arranged in two V-shaped blocks, and the PET and CT components are assembled on a rotating gantry in such a way that there is no axial displacement between the geometric centres of the two modalities. With 2-D FBP (as per NEMA NU 4 standards), the spatial resolution of this system is 1.48 mm at the centre of the FOV, and 1.81 mm and 2.14 mm at 2 cm and 3 cm radial offsets, respectively. Illustrations of the PETbox and VrPET/CT systems are presented in Fig. 6.

### Perspectives related to technological evolution

**Fig. 7:**
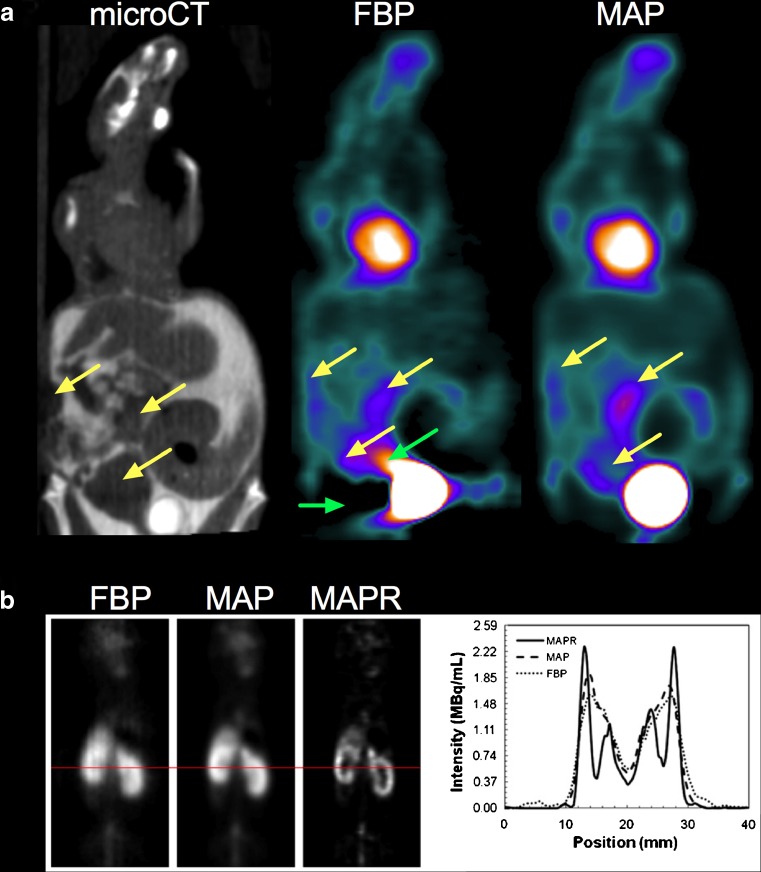
Technological improvements that could benefit high-throughput PET imaging. **a** A tumour-bearing mouse imaged in a group of four animals in the customized bed shown in Fig. 3. Animals received intravenous and intraperitoneal injections of iodinated contrast medium. Images were reconstructed with FBP and MAP reconstruction, an algorithm that models the PSF of the system. Images are scaled to the same maximum. Note the artefacts near the bladder on the FBP images, which hamper the detection of a necrotic tumour also located near the bladder. These artefacts are not present on the MAP images. **b** Mice imaged using ^61^Cu-PTSM. Relative to that obtained with FBP, improved image resolution was achieved by the use of MAP reconstruction with PSF and positron range modelling (MAPR). With MAPR, the renal cortex can be resolved clearly in the kidney. **b** reprinted with permission of the Society of Nuclear Medicine from de Kemp et al. [19]

High-throughput imaging of rodents will benefit from ongoing hardware evolution such as new detectors capable of achieving submillimetre spatial resolution and higher sensitivity [46] and advanced reconstruction algorithms modelling the PSF of the system or including both PSF and positron range modelling [19]. An example of an advanced reconstruction algorithm is shown in Fig. 7.

### Issues related to animal handling

A potential issue when developing high-throughput SA-PET strategies relates to animal handling and anaesthesia as well as efficient coordination of tracer injection. Anaesthesia is generally obtained using isoflurane inhalation, and devices allowing up to six mice to be kept under anaesthesia are commercially available. Ideally, customized beds should include heated covers, body temperature probes and respiratory pads, as long periods of anaesthesia can lead to a significant decrease in body temperature, which can in turn induce morbidity and modifications of tracer uptake, as shown for ^18^F-FDG [9] and ^18^F-FLT [10], presumably because of decreased blood flow and decreased body functions at the lower temperature. Ketamine/xylazine anaesthesia should be avoided as it has been shown to induce marked hyperglycaemia in mice [47]. Extreme hyperglycaemia (>500 mg/dl) has been shown by Wahl et al. [48] to reduce significantly ^18^F-FDG uptake in rat xenografts, which suggests competition between glucose and ^18^F-FDG. It is noteworthy that isoflurane may also affect glucose blood levels, but conflicting results have been reported with either a variable decrease or a slight increase [47] in glucose blood levels. In their study comparing the impact of isoflurane and sevoflurane on blood glucose levels in various mouse strains and implanted tumours, Flores et al. [49] showed that sevoflurane may be preferred to isoflurane in mice undergoing small-animal imaging, as blood glucose levels were more stable under sevoflurane than under isoflurane, especially in nude mice. Sevoflurane also has advantages such as fewer hepatotoxic metabolites and a more rapid induction and recovery profile, which could be useful, particularly in fragile mouse models.

Although no guidelines on SA-PET imaging in cancer research have been published so far, EANM guidelines on PET tumour imaging [50] provide insights into issues related to imaging of multiple mice. For therapy monitoring in oncology, EANM guidelines recommend that the delay between tracer injection and image acquisition is the same (± 5 min) between longitudinal studies. Obviously, a similar approach should be used for animal imaging. In our experience, injecting four mice within 5 min is feasible for two well-trained researchers working side by side. When performed by experienced researchers, variability in the efficiency of tail vein injection does not affect %ID/g: extravasation of tracer at the site of injection has been shown to occur quite rarely (in 4 of 23 scans [11] and in 4 of 34 scans [10]), has never exceeded 10 % of the injected dose, and correction of %ID/g by subtracting the tail vein activity from the injected dose has been shown to induce only a slight increase in %ID/g [10, 11]. Another option is to determine when the plateau phase is reached for a specific animal model and a given tracer. This can be ascertained from dynamic scans. If imaged after this time, variations in the delay between tracer injection and imaging will minimally affect tumour tracer uptake.

 Also feasible is intraperitoneal (IP) injection of the PET probe, which is a more convenient and rapid technique for animal handlers than is gaining intravenous (IV) access. This technique has been shown to lead to similar radioactivity concentrations in liver and tumour in mice bearing glioma implanted subcutaneously 60 min following ^18^F-FDG injection compared to IV administration [47]. These findings were later confirmed by Wong et al., who showed that IV and IP injections provide equivalent pharmacokinetic parameters and comparable ^18^F-FDG biodistribution 60 min after injection [51]. This injection route was also used by Revheim et al. [29] for imaging ten mice simultaneously on a clinical PET/CT system. A potential drawback of IP injection is that the initial distribution of IP-injected ^18^F-FDG in various organs has been reported to be slower than that following IV injection because ^18^F-FDG diffuses via the peritoneal membrane and is subsequently absorbed via the portal system, thus indicating that IP injection is not suitable for tracers eliminated primarily by the liver [51].

## What throughput could be achieved and what would be the consequences on the use of PET by the nuclear medicine and oncology communities?

The gain in throughput in a working day is likely to be higher for large-bore dedicated SA-PET systems, as compared to high-resolution clinical PET/CT systems on which preclinical research would usually be performed on an after-hours basis, and planar positron imaging systems. Indeed, taking the example of the customized bed illustrated in Fig. 2, four mice can be imaged in 25 min (15 min for the emission and 10 min for the CT acquisition) as opposed to approximately 120 min if the animals had been imaged singly (taking into account induction of anaesthesia and positioning of the animals in the SA-PET system). This 4.8-fold gain in throughput would help researchers to meet the statistical requirements of experiments requiring a large number of animals to be imaged, or in biodistribution studies, especially with short-lived positron emitters, since multiple experiments could be performed using a single synthesis, thus dramatically improving the cost-effectiveness of SA-PET imaging. More importantly, high-throughput SA-PET imaging, performed within the framework of translational research (i.e. together with a full range of molecular biology techniques) is likely to significantly enhance the use of PET Imaging in phase 1 trials.

 Tseng et al. [52] recently reported a strategy based on the use of DNA microarray analysis and SA-PET imaging as complementary technologies in drug development. These authors proposed a stepwise approach to selecting a suitable imaging tracer for tumour evaluation. Gene expression analysis is first performed on tumour cells treated with drugs in vitro either by means of DNA microarray or with quantitative polymerase chain reaction interrogating a panel of key genes representing biological pathways relevant to imaging. Pathways that are most affected by treatment suggest PET probes that may subsequently be used in tracer accumulation studies in cell culture and SA-PET studies in mice bearing human xenografted tumours. Tseng et al. [52] validated their stepwise approach in melanoma cells treated with RAF265, a novel B-B-Raf/VEGFR-2 inhibitor, which causes a decrease in ^18^F-FDG uptake as early as 1 day after treatment initiation, thus supporting the use of ^18^F-FDG PET in phase 1 clinical trials using RAF265. In the case of molecularly targeted therapies for which the OBD or optimal scheduling are not known, the stepwise approach described by Tseng et al. [52], together with high-throughput SA-PET imaging using appropriate probes, would optimize the use of PET technology in cancer research from bench to bedside by reducing costs and saving time. The relevance of these approaches has recently been demonstrated by the demonstration of a marked early reduction in ^18^F-FDG uptake in BRAF mutant melanoma using the novel BRAF inhibitor, vemurafenib [53].

## Conclusion

There are many potential benefits to be derived from high-throughput SA-PET imaging. Further, technical and design innovations offer the potential to ensure that the process of evaluation of new therapies is more efficient. This will reduce the time and cost of using molecular imaging in drug development and provide greater impetus for the integration of PET into early phase human trials of novel targeted therapies.

## References

[1] Adjei AA (2006). What is the right dose? The elusive optimal biologic dose in phase I clinical trials. J Clin Oncol.

[2] Le Tourneau C, Lee JJ, Siu LL (2009). Dose escalation methods in phase I cancer clinical trials. J Natl Cancer Inst.

[3] Rojo F, Dalmases A, Corominas JM, Albanell J (2007). Pharmacodynamics: biological activity of targeted therapies in clinical trials. Clin Transl Oncol.

[4] Fuereder T, Wanek T, Pflegerl P, Jaeger-Lansky A, Hoeflmayer D, Strommer S (2011). Gastric cancer growth control by BEZ235 in vivo does not correlate with PI3K/mTOR target inhibition but with [18F]FLT uptake. Clin Cancer Res.

[5] Willmann JK, van Bruggen N, Dinkelborg LM, Gambhir SS (2008). Molecular imaging in drug development. Nat Rev Drug Discov.

[6] Cejka D, Kuntner C, Preusser M, Fritzer-Szekeres M, Fueger BJ, Strommer S (2009). FDG uptake is a surrogate marker for defining the optimal biological dose of the mTOR inhibitor everolimus in vivo. Br J Cancer.

[7] Eckelman WC (2008). Further discussions on choosing the number of animals for an experiment. Nucl Med Biol.

[8] Eckelman WC, Kilbourn MR, Joyal JL, Labiris R, Valliant JF (2007). Justifying the number of animals for each experiment. Nucl Med Biol.

[9] Dandekar M, Tseng JR, Gambhir SS (2007). Reproducibility of 18F-FDG microPET studies in mouse tumor xenografts. J Nucl Med.

[10] Tseng JR, Dandekar M, Subbarayan M, Cheng Z, Park JM, Louie S (2005). Reproducibility of 3'-deoxy-3'-(18)F-fluorothymidine microPET studies in tumor xenografts in mice. J Nucl Med.

[11] Chang E, Liu S, Gowrishankar G, Yaghoubi S, Wedgeworth JP, Chin F (2011). Reproducibility study of [(18)F]FPP(RGD)2 uptake in murine models of human tumor xenografts. Eur J Nucl Med Mol Imaging.

[12] Aide N, Kinross K, Cullinane C, Roselt P, Waldeck K, Neels O (2010). 18F-FLT PET as a surrogate marker of drug efficacy during mTOR inhibition by everolimus in a preclinical cisplatin-resistant ovarian tumor model. J Nucl Med.

[13] Cullinane C, Dorow DS, Jackson S, Solomon B, Bogatyreva E, Binns D (2011). Differential (18)F-FDG and 3'-deoxy-3'-(18)F-fluorothymidine PET responses to pharmacologic inhibition of the c-MET receptor in preclinical tumor models. J Nucl Med.

[14] Aide N, Desmonts C, Beauregard JM, Beyer T, Kinross K, Roselt P (2010). High throughput static and dynamic small animal imaging using clinical PET/CT: potential preclinical applications. Eur J Nucl Med Mol Imaging.

[15] Disselhorst JA, Brom M, Laverman P, Slump CH, Boerman OC, Oyen WJ (2010). Image-quality assessment for several positron emitters using the NEMA NU 4-2008 standards in the Siemens Inveon small-animal PET scanner. J Nucl Med.

[16] Liu X, Laforest R (2009). Quantitative small animal PET imaging with nonconventional nuclides. Nucl Med Biol.

[17] Anizan N, Carlier T, Hindorf C, Barbet J, Bardies M (2012). Acquisition setting optimization and quantitative imaging for 124I studies with the Inveon microPET-CT system. EJNMMI Res.

[18] Ruangma A, Bai B, Lewis JS, Sun X, Welch MJ, Leahy R (2006). Three-dimensional maximum a posteriori (MAP) imaging with radiopharmaceuticals labeled with three Cu radionuclides. Nucl Med Biol.

[19] de Kemp RA, Epstein FH, Catana C, Tsui BM, Ritman EL (2010). Small-animal molecular imaging methods. J Nucl Med.

[20] Tatsumi M, Nakamoto Y, Traughber B, Marshall LT, Geschwind JF, Wahl RL (2003). Initial experience in small animal tumor imaging with a clinical positron emission tomography/computed tomography scanner using 2-[F-18]fluoro-2-deoxy-D-glucose. Cancer Res.

[21] Seemann MD, Beck R, Ziegler S (2006). In vivo tumor imaging in mice using a state-of-the-art clinical PET/CT in comparison with a small animal PET and a small animal CT. Technol Cancer Res Treat.

[22] Brix G, Doll J, Bellemann ME, Trojan H, Haberkorn U, Schmidlin P (1997). Use of scanner characteristics in iterative image reconstruction for high-resolution positron emission tomography studies of small animals. Eur J Nucl Med.

[23] Aide N, Kinross K, Beauregard JM, Neels O, Potdevin T, Roselt P (2011). A dual radiologic contrast agent protocol for 18F-FDG and 18F-FLT PET/CT imaging of mice bearing abdominal tumors. Mol Imaging Biol.

[24] DiFilippo FP, Patel S, Asosingh K, Erzurum SC (2012). Small-animal imaging using clinical positron emission tomography/computed tomography and super-resolution. Mol Imaging.

[25] Jakoby BW, Bercier Y, Conti M, Casey ME, Bendriem B, Townsend DW (2011). Physical and clinical performance of the mCT time-of-flight PET/CT scanner. Phys Med Biol.

[26] Bao Q, Newport D, Chen M, Stout DB, Chatziioannou AF (2009). Performance evaluation of the inveon dedicated PET preclinical tomograph based on the NEMA NU-4 standards. J Nucl Med.

[27] Szanda I, Mackewn J, Patay G, Major P, Sunassee K, Mullen GE (2011). National Electrical Manufacturers Association NU-4 performance evaluation of the PET component of the NanoPET/CT preclinical PET/CT scanner. J Nucl Med.

[28] Prasad R, Ratib O, Zaidi H (2010). Performance evaluation of the FLEX triumph X-PET scanner using the national electrical manufacturers association NU-4 standards. J Nucl Med.

[29] Revheim ME, Roe K, Bruland OS, Bach-Gansmo T, Skretting A, Seierstad T (2011). Monitoring the effect of targeted therapies in a gastrointestinal stromal tumor xenograft using a clinical PET/CT. Mol Imaging Biol.

[30] Visser EP, Disselhorst JA, Brom M, Laverman P, Gotthardt M, Oyen WJ (2009). Spatial resolution and sensitivity of the Inveon small-animal PET scanner. J Nucl Med.

[31] Paproski RJ, Wuest M, Jans H, Graham K, Gati W, McQuarrie S (2010). Biodistribution and uptake of 3'-deoxy-3'-fluorothymidine in ENT1-knockout mice and in an ENT1-knockdown tumor model. J Nucl Med.

[32] Aide N, Desmonts C, Briand M, Meryet-Figuiere M, Poulain L (2010). High-throughput small animal PET imaging in cancer research: evaluation of the capability of the Inveon scanner to image four mice simultaneously. Nucl Med Commun.

[33] Siepel FJ, van Lier MG, Chen M, Disselhorst JA, Meeuwis AP, Oyen WJ (2010). Scanning multiple mice in a small-animal PET scanner: influence on image quality. Nucl Instrum Methods Phys Res A.

[34] Chow PL, Rannou FR, Chatziioannou AF (2005). Attenuation correction for small animal PET tomographs. Phys Med Biol.

[35] Jensen MM, Jorgensen JT, Binderup T, Kjaer A (2008). Tumor volume in subcutaneous mouse xenografts measured by microCT is more accurate and reproducible than determined by 18F-FDG-microPET or external caliper. BMC Med Imaging.

[36] Kersemans V, Thompson J, Cornelissen B, Woodcock M, Allen PD, Buls N (2011). Micro-CT for anatomic referencing in PET and SPECT: radiation dose, biologic damage, and image quality. J Nucl Med.

[37] Huisman MC, Reder S, Weber AW, Ziegler SI, Schwaiger M (2007). Performance evaluation of the Philips MOSAIC small animal PET scanner. Eur J Nucl Med Mol Imaging.

[38] Prasad R, Ratib O, Zaidi H (2011). NEMA NU-04-based performance characteristics of the LabPET-8 small animal PET scanner. Phys Med Biol.

[39] Chatziioannou A, Qi J, Moore A, Annala A, Nguyen K, Leahy R (2000). Comparison of 3-D maximum a posteriori and filtered backprojection algorithms for high-resolution animal imaging with microPET. IEEE Trans Med Imaging.

[40] Qi J, Leahy RM, Cherry SR, Chatziioannou A, Farquhar TH (1998). High-resolution 3D Bayesian image reconstruction using the microPET small-animal scanner. Phys Med Biol.

[41] Visser EP, Disselhorst JA, van Lier MGTB, Laverman P, de Jong GM, Oyen WJ (2011). Characterization and optimization of image quality as a function of reconstruction algorithms and parameter settings in a Siemens Inveon small-animal PET scanner using the NEMA NU4-2008 standards. Nucl Instrum Methods Phys Res A.

[42] Takamatsu H, Kakiuchi T, Noda A, Uchida H, Nishiyama S, Ichise R (2004). An application of a new planar positron imaging system (PPIS) in a small animal: MPTP-induced parkinsonism in mouse. Ann Nucl Med.

[43] Uchida H, Sato K, Kakiuchi T, Fukumoto D, Tsukada H (2008). Feasibility study of quantitative radioactivity monitoring of tumor tissues inoculated into mice with a planar positron imaging system (PPIS). Ann Nucl Med.

[44] Zhang H, Bao Q, Vu NT, Silverman RW, Taschereau R, Berry-Pusey BN (2010). Performance evaluation of PETbox: a low cost bench top preclinical PET scanner. Mol Imaging Biol.

[45] Lage E, Vaquero JJ, Sisniega A, Espana S, Tapias G, Abella M (2009). Design and performance evaluation of a coplanar multimodality scanner for rodent imaging. Phys Med Biol.

[46] Levin CS (2012). Promising new photon detection concepts for high-resolution clinical and preclinical PET. J Nucl Med.

[47] Fueger BJ, Czernin J, Hildebrandt I, Tran C, Halpern BS, Stout DB (2006). Impact of animal handling on the results of 18F-FDG PET studies in mice. J Nucl Med.

[48] Wahl RL, Henry CA, Ethier SP (1992). Serum glucose: effects on tumor and normal tissue accumulation of 2-[F-18]-fluoro-2-deoxy-D-glucose in rodents with mammary carcinoma. Radiology..

[49] Flores JE, McFarland LM, Vanderbilt A, Ogasawara AK, Williams SP (2008). The effects of anesthetic agent and carrier gas on blood glucose and tissue uptake in mice undergoing dynamic FDG-PET imaging: sevoflurane and isoflurane compared in air and in oxygen. Mol Imaging Biol.

[50] Boellaard R, O'Doherty MJ, Weber WA, Mottaghy FM, Lonsdale MN, Stroobants SG (2010). FDG PET and PET/CT: EANM procedure guidelines for tumour PET imaging: version 1.0. Eur J Nucl Med Mol Imaging.

[51] Wong KP, Sha W, Zhang X, Huang SC (2011). Effects of administration route, dietary condition, and blood glucose level on kinetics and uptake of 18F-FDG in mice. J Nucl Med.

[52] Tseng JR, Stuart D, Aardalen K, Kaplan A, Aziz N, Hughes NP (2011). Use of DNA microarray and small animal positron emission tomography in preclinical drug evaluation of RAF265, a novel B-Raf/VEGFR-2 inhibitor. Neoplasia.

[53] McArthur GA, Puzanov I, Amaravadi R, Ribas A, Chapman P, Kim KB (2012). Marked, homogeneous and early FDG-PET responses to vemurafenib in BRAF-mutant advanced melanoma. J Clin Oncol.

